# Postoperative Adjuvant Therapy for Patients with pN+ Esophageal Squamous Cell Carcinoma

**DOI:** 10.1155/2021/8571438

**Published:** 2021-01-22

**Authors:** Juan Li, Rong Qiu, Yuanping Hu, Yuxiang Wang, Zhan Qi, Ming He, Yuekao Li

**Affiliations:** ^1^Department of Radiation Oncology, Fourth Hospital of Hebei Medical University & Hebei Clinical Research Center for Radiation Oncology, Shijiazhuang 050011, China; ^2^Department of Thoracic Surgery, Fourth Hospital of Hebei Medical University, Shijiazhuang 050011, China; ^3^Department of CT/MRI, Fourth Hospital of Hebei Medical University, Shijiazhuang 050011, China

## Abstract

Esophageal squamous cell carcinoma (ESCC) is the most common pathological type of esophageal cancer in China. However, patient survival time after surgery remains unsatisfactory, especially in those who are pN+. This retrospective study determined the value of postoperative adjuvant therapy for patients with pN+ ESCC. From Jan 2008 to Sep 2011, 453 pN+ ESCC patients who underwent R0 resection and survived for at least 1 month were retrospectively enrolled. All patients received surgery. Some patients received surgery alone (SA, *n* = 131), and others received postoperative chemotherapy (POCT, *n* = 222), radiotherapy (PORT, *n* = 57), or sequential chemoradiotherapy (POCRT, *n* = 43). The follow-up ended on 1 Dec 2019. The 5-year overall survival (OS), disease-free survival (DFS), and locoregional recurrence (LR) were significantly worse in the SA group (15.2%, 13.1%, and 71.6%, all *p* < 0.05) than in the POCT group (28.0%, 20.8%, and 66.5%), the PORT group (27.4%, 24.4%, and 46.9%), and the POCRT group (42.8%, 35.5%, and 43.0%). Furthermore, compared with the SA group, the median OS and DFS were significantly longer in the POCT, PORT, and POCRT groups (all *p* < 0.05). PORT and POCRT (but not POCT) also significantly reduced the LR (*p* < 0.01). Multivariate Cox analysis showed that each type of postoperative therapy was independently associated with improvements in OS, DFS, and LR. Postoperative adjuvant therapy—either POCT, PORT, or POCRT—significantly improved OS and DFS in patients with pN+ ESCC after R0 surgery. PORT and PORCT significantly reduced LR in these patients.

## 1. Introduction

Bray et al. [1] estimated that esophageal cancer (EC) was the seventh most common cancer and the sixth leading cause of cancer deaths worldwide. Chen et al. [2] ranked EC as fifth in incidence and fourth in mortality among all cancers in China. Esophageal squamous cell carcinoma (ESCC) is the most common pathological type of EC in Asia (especially China), although esophageal adenocarcinoma is more common in Western countries [3]. The standard primary treatment recommended by the National Comprehensive Cancer Network (NCCN) guidelines for resectable ESCC is neoadjuvant chemoradiotherapy followed by surgery [4]. However, many patients receive surgery first, and this is still an option in the guidelines. For patients who received R0 resections, the NCCN guidelines only recommend a regular follow-up.

However, the postoperative survival time of patients with ESCC remains unsatisfactory, especially for those with positive lymph node metastasis (pN+) [5–10]. Previous studies reported that postoperative radiotherapy (PORT), postoperative chemoradiotherapy (POCRT), and even postoperative chemotherapy (POCT) improved survival in patients with locally advanced ESCC [5–14]. Based on our previous research [6, 7], we retrospectively reevaluated the value of different postoperative treatments on the long-time survival of patients with pN+ ESCC after R0 resection.

## 2. Materials and Methods

### 2.1. Eligibility

Each included patient had radical esophagectomy with two- or three-field lymphadenectomy between Jan 2009 and Dec 2011, pathologically confirmed ESCC, pathologically confirmed pN+ and pT2/4, no history of another tumor, Karnofsky performance status of 70 or more, survival time of 1 month or more, and received follow-up examinations in our previous studies [6, 7]. This study was approved by the Medical Ethics Committee of our hospital, and each included individual signed an informed consent agreement. Pathology and staging were according to the 7^th^ TNM cancer staging criteria.

### 2.2. Surgery

Left thoracotomy was the most common surgical approach for patients with middle- and lower-thoracic ESCC, and right thoracotomy was the most common approach for patients with upper-thoracic ESCC. Most patients accepted two-field lymphadenectomy. Some upper-thoracic ESCC patients accepted three-field lymphadenectomy.

### 2.3. Postoperative Adjuvant Therapy

The NCCN guidelines for ESCC patients with R0 resection only recommend a regular follow-up. Thus, postoperative adjuvant therapy was not performed for all patients, mainly based on the surgeon's recommendation and each patient's physical and financial status. POCT consisted of cisplatin with fluorouracil or paclitaxel/docetaxel, and these patients received a median of 3 cycles (range: 1–6). Postoperative radiotherapy (PORT) was initiated 4 to 8 weeks after surgery and consisted of three-dimensional conformal radiotherapy (3D-CRT) or intensity-modulated radiotherapy (IMRT). The clinical target volume (CTV) was the upper mediastinum, supraclavicular, and lower neck area for patients with upper thoracic ESCC; the whole mediastinum, with or without the supraclavicular area, for patients with middle thoracic ESCC; and the middle and lower mediastinum and gastric left lymphatic drainage area for patients with lower ESCC. The total dosage was 50 to 54 Gy, and there were 25 to 28 fractions, 1.8 to 2.0 Gy/fraction, and 5 fractions per week. Chemoradiotherapy (POCRT) consisted of the sequential combination of PORT and POCT.

### 2.4. Follow-Up

The follow-up ended on 1 Dec 2019. Patients were instructed to return for follow-up evaluations every 3 months for the first two years, every 6 months for the next three years. The procedures performed at each follow-up were computed tomography (CT) with contrast of the neck, thorax, and upper abdomen; ultrasonography of the neck and upper abdomen; nuclear bone scanning; conventional blood and biochemistry studies; and gastric endoscopy, positron emission tomography, and cytologic puncture (if needed).

The long-term outcomes were determined by review of the medical records and follow-up data. Overall survival (OS) was considered to be the time from the operation to death or the last follow-up, and disease-free survival (DFS) as the time from the operation to first disease failure (locoregional recurrence, distant metastasis, combined recurrence, or death from any cause). Locoregional recurrence (LR) was based on the positivity of the primary esophageal tumor bed, anastomotic sites, or regional lymph nodes (LNs; supraclavicular, mediastinal, and celiac axis LNs). Recurrence beyond those sites was considered distant metastasis (DM).

### 2.5. Statistical Analysis

All statistical analyses were conducted using SPSS version 22.0 (IBM Corp., Armonk, NY, USA). OS, DFS, and LR were calculated using Kaplan-Meier analysis, and groups were compared using the log-rank test. Univariate and multivariate analyses were performed using a Cox proportional hazard regression model. A *p* value below 0.05 was considered significant.

## 3. Results

### 3.1. Comparison of Patients Who Received and Did Not Receive Adjuvant Therapy

We enrolled 453 eligible patients who had pN+ ESCC (Table 1). The median age was 60 years (range: 37–83), the median lesion length was 6 cm (range: 1–16), and the median number of resected lymph nodes (LNs) was 10 (range: 1–34). A total of 131 patients received surgery alone (SA), 222 received POCT, 57 received PORT, and 43 received POCRT.

Our comparison of the clinicopathological factors of patients who did and did not receive postoperative adjuvant therapy indicated that there were significant differences in age, resected LNs, pT stage, pTNM stage, and tumor cell differentiation (all *p* < 0.05), but no significant differences in gender, site of lesion, length of lesion, pN stage, and pTNM stage (all *p* > 0.05). Thus, patients who received postoperative adjuvant therapy were more likely to be young, have 12 or more resected LNs, have pT3 stage, and have poor tumor cell differentiation.

### 3.2. Overall Survival

**Figure 1:**
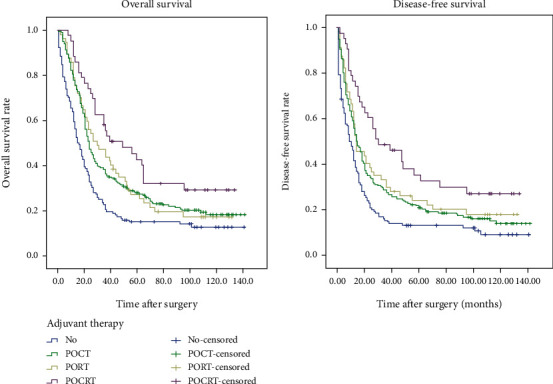
Overall survival (a) and disease-free survival (b) of patients who received surgery alone (SA) or different types of adjuvant therapy.

At the end of the second follow-up period, the rate of follow-up was 94.5%. A total of 363 of 453 patients (83.4%) died, 310 from tumor relapse and 53 from nonneoplastic causes. Kaplan-Meier analysis (Figure 1(a)) showed that the OS in the SA group was 59.6% at 1 year, 20.6% at 3 years, and 15.2% at 5 years, and the median OS was 16 months. The OS in the POCT group was 77.9% at 1 year, 36.5% at 3 years, and 28.0% at 5 years, and the median OS was 24 months. The OS in the PORT group was 78.9% at 1 year, 43.9% at 3 years, and 27.4% at 5 years, and the median OS was 30 months. The OS in the POCRT group was 88.4% at 1 year, 55.7% at 3 years, and 42.8% at 5 years, and the median OS was 49 months. Subgroup analysis indicated that OS was significantly different between the SA and POCT groups, SA and PORT groups, SA and POCRT groups, and POCT and POCRT groups (all *p* < 0.05). However, OS was not significantly different between the POCT and PORT groups and the PORT and POCRT groups (both *p* > 0.05).

Relative to patients in the SA group, those who received any type of adjuvant therapy had a significantly better 5-year OS (29.9% *vs.* 15.2%) and a longer median OS (26 months, IQR: 22.5–29.4 *vs.* 16 months, IQR: 12.5–19.3 months; *p* < 0.001). Subgroup analysis (data not shown) indicated that adjuvant therapy significantly increased OS in patients regardless of age, lesion length, number of dissected lymph nodes, and pN stage and in those who were male, with middle-segment tumors, with stage pT3, and with TNM stage III (all *p* < 0.05). Adjuvant therapy did not improve the OS of females, those with tumors in the upper or lower segment, those with stage pT2 or pT4, or those with TNM stage IIb (all *p* > 0.05).

### 3.3. Disease-Free Survival

Kaplan-Meier analysis (Figure 1(b)) showed that the DFS in the SA group was 41.7% at 1 year, 15.4% at 3 years, and 13.1% at 5 years, and the median DFS was 10 months. The DFS in the POCT group was 55.9% at 1 year, 27.5% at 3 years, and 20.8% at 5 years, and the median DFS was 14 months. The DFS in the PORT group was 57.9% at 1 year, 29.8% at 3 years, and 24.4% at 5 years, and the median DFS was 14 months. The DFS in the POCRT group was 76.7% at 1 year, 48.8% at 3 years, and 35.5% at 5 years, and the median DFS was 30 months. Subgroup analysis indicated that DFS was significantly different between the SA and POCT groups, SA and PORT groups, SA and POCRT groups, and POCT and POCRT groups (all *p* < 0.05). However, DFS was not significantly different between the POCT and PORT groups and the PORT and POCRT groups (both *p* > 0.05).

Relative to patients in the SA group, those who received any type of adjuvant therapy had significantly better 5-year DFS (23.4% *vs.* 31.1%) and a longer median OS (16 months, IQR: 13.1–18.9 *vs.* 10 months, IQR: 7.2–12.8; *p* < 0.001). Subgroup analysis (data not shown) indicated that adjuvant therapy significantly increased DFS in patients who had dissected LNs, tumor lesions less than 6 cm, upper- or middle-segment tumors, stage pT3, and TNM stage IIIa/b stage pN and in those who were male and younger than 60 years (all *p* < 0.05). Adjuvant therapy had no impact on the DFS of patients who were female, older than 60 years, had lesions of 6 cm or more, had lower-segment lesions, had stage pT2 or pT4, and had TNM stage IIb or IIIc (all *p* > 0.05).

### 3.4. Locoregional Recurrence

**Figure 2:**
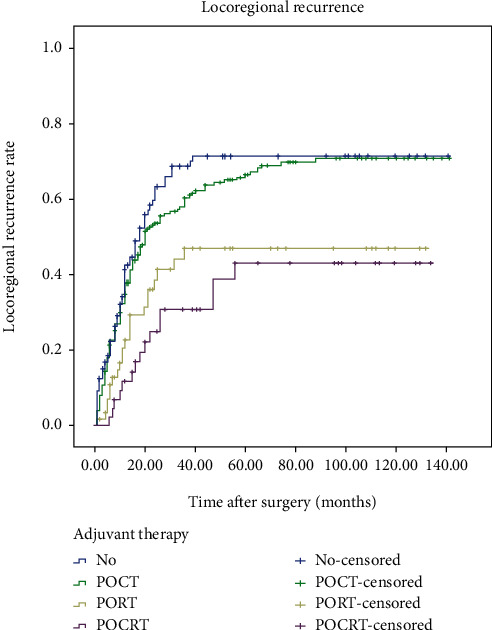
Locoregional recurrence of patients who received surgery alone (SA) or different types of adjuvant therapy.

At the end of follow-up, 245 patients had LR (Figure 2). Kaplan-Meier analysis showed that the LR rate in the SA group was 41.3% at 1 year, 68.4% at 3 years, and 71.6% at 5 years. The LR rate in the POCT group was 34.8% at 1 year, 60.4% at 3 years, and 66.5% at 5 years. The LR rate in the POCRT group was 22.7% at 1 year, 46.9% at 3 years, and 46.9% at 5 years. These four survival curves were significantly different (*p* < 0.001). Furthermore, compared with the SA group, the LR was significantly reduced in the PORT group (*p* = 0.003) and the POCRT group (*p* < 0.001), but not in the POCT group (*p* > 0.05).

### 3.5. Cox Analysis of OS, DFS, and LR

We also performed univariate and multivariate Cox analyses to determine the effect of 9 factors (gender, age, site of lesion, length of tumor, surgery (2 *vs.* 3 fields), dissected LN, differentiation of squamous cell carcinoma, pTNM stage, and postoperative adjuvant therapy) on the 3 outcome measures. The results showed that OS was independently associated with gender, site of lesion, number of resected LNs, pTNM stage, and receipt of postoperative adjuvant therapy (Table 2). DFS and LR were independently associated with the site of lesion, pTNM stage, and postoperative adjuvant therapy (Table 3). Univariate and multivariate COX analyses also showed that none of the analyzed factors was associated with DM (data not shown).

## 4. Discussion

We retrospectively examined patients with pN+ ESCC after R0 resection. One of our most notable findings was that POCT, relative to SA, significantly improved the 5-year OS from 15.2% to 28.0% and the 5-year DFS from 13.1% to 20.8%. Numerous other studies also examined the effect of POCT in patients with pN+ ESCC after resection. Two prospective studies showed that POCT improved DFS but not OS in patients with ESCC [11, 12]. Qin et al. [14] showed that POCT (docetaxel- or paclitaxel-based regimens) improved the 5-year DFS from 20.2% to 34% and potentially prolonged OS in patients with pN+ ESCC. In contrast, Xu et al. [5] showed that POCT did not improve the OS in patients with pN+ ESCC. Zhang et al. [13] examined patients with pN+ ESCC and found that POCT (4–6 cycles of paclitaxel and cisplatin), relative to SA, increased the 3-year DFS from 34.6% to 56.3% and the 3-year OS from 37.5% to 55.0%. Li et al. [15] showed that POCT, relative to SA, increased the 3-year DFS from 19.9% to 41.6% and the 3-year OS from 30.8% to 53.7% in patients with stage pN2/3 ESCC, but this treatment did not improve outcomes for patients with pN1 ESCC. Sohda et al. and our previous study also showed that POCT improved OS compared with SA [16, 17]. A meta-analysis by Zhao et al. [18] identified POCT as an independent factor associated with favorable prognosis in patients with ESCC, because it improved OS and DFS. These results are similar to our results [13–18] and thus support the use of POCT to increase survival in patients with pN+ ESCC.

The present study also demonstrated that PORT significantly improved OS and DFS. Similarly, Ni et al. [8] showed that PORT, relative to SA, increased the 5-year OS from 31.3% to 45.0% and the 5-year DFS from 24.2% to 39.8% in patients with pN+ ESCC. Zhang et al. [19] showed that PORT *via* IMRT improved the 5-year OS and DFS of patients with TNM stage IIb and III ESCC. Several other reports also showed that PORT, rather than SA, improved OS in patients with pN+ or stage III ESCC [5, 20–22]. A systemic review by Liu et al. [23] showed that PORT significantly improved DFS and OS in patients with pN+ ESCC. Yu et al. [10] showed that the 5-year OS was significantly better in patients who received preoperative RT rather than PORT for those with TNM stage III ESCC, but not for those with TNM stage II ESCC. Taken together, these results show that PORT can significantly improve survival in patients with pN+ ESCC.

We also found that sequential POCRT significantly improved OS and DFS compared with SA. In agreement, Li et al. [15] reported that POCRT for patients with pN+ ESCC increased the 3-year DFS from 19.9% to 34.0% and the 3-year OS from 30.8% to 50.5%. The results of Wong et al. [24] indicated that the addition of POCRT (administered sequentially or concomitantly) for patients with pN+ EC was associated with improved OS.

Our results also showed that the OS and DFS were not significantly different for patients with pN+ ESCC who received PORT or POCRT. These results are similar to those of Lu et al. [25], Zhang et al. [26], and Yu et al. [22]. In contrast, Zhang et al. [26] examined patients with TNM stage II/III ESCC and showed that the OS was significantly worse for those who received sequential POCRT rather than PORT or concurrent POCRT. Zou et al. [27], Hsu et al. [28], and Song et al. [9] showed that POCRT significantly increased OS and DFS compared with PORT in patients with pN+ ESCC. The present study also found that POCRT significantly improved OS (*p* = 0.034) and DFS (*p* = 0.009) compared with POCT. In contrast, Li et al. [15] showed no notable differences in OS and DFS following POCT and POCRT in patients with pN+ ESCC.

We found that postoperative adjuvant therapy, relative to SA, significantly increased the 5-year OS from 15.2% to 29.9% and the 5-year DFS from 13.3% to 23.4%. In addition, our subgroup analyses found that adjuvant therapy significantly increased OS in males, and in patients with middle-segment tumors, stage pT3, stage pN1/3, or TNM stage III. Yu et al. [22] showed that adjuvant therapy significantly increased the 5-year OS from 26.4% to 37.1%, and Li et al. [15] showed that POCT and POCRT improved the OS and DFS in patients with pN2/3 ESCC or ESCC in the middle thoracic region, but not for those with pN1 ESCC or ESCC in the lower thoracic region. Ni et al. [8] showed that PORT, relative to SA, significantly improved the 5-year OS of patients with pT3/4N1M0 ESCC from 23.5% to 41.3%, but did not improve the 5-year OS of patients with pT1/2N1M0 ESCC.

Our findings also indicated that PORT and POCRT, relative to SA, significantly decreased LR, but POCT provided no benefit. In agreement, the results of Ni et al. [8] indicated that PORT significantly decreased LR, with an absolute difference of about 26% in patients with pN+ ESCC, including recurrence in the supraclavicular and mediastinal regions. Zou et al. [27] showed that POCRT for patients with pN+ ESCC significantly increased the median local recurrence-free survival from 21 to 42 months compared with SA. Chen et al. [20] found that PORT was also associated with lower recurrence rates in patients with tumors in the supraclavicular and upper- and middle-mediastinal regions. Zeng et al. [29] found that PORT reduced the LR from 92.0% to 35.7% in patients with TNM stage II/III ESCC. A systemic review by Liu et al. [23] concluded that PORT clearly reduced the risk of LR in patients with ESCC. Song et al. [9] reported that POCRT, relative to PORT, administered to patients with pN+ ESCC significantly decreased DM. In contrast, our results and the results of Zou et al. [27] indicated no significant differences in recurrence or DM in groups that received POCRT and PORT.

To our knowledge, this is the first single-center retrospective study to compare SA and surgery with three different types of postoperative adjuvant therapy for patients with pN+ ESCC. The results showed that adjuvant therapy—either POCT, PORT, or POCRT—significantly increased OS and DFS compared with SA. Furthermore, PORT and POCRT reduced LR, although POCT had no impact on LR. Thus, adjuvant therapy should be recommended for these patients. Because survival was highest in the POCRT group, patients should receive POCRT if it can be tolerated.

This study has several limitations. First, because this was a retrospective single-center study, the possibility of selection bias cannot be excluded. Second, the current NCCN guidelines recommend neoadjuvant therapy for node-positive ESCC. But in China, many ESCC patients select surgery first, and this regimen is also recommended by the Chinese guidelines. Based on the surgeon's recommendation and each patient's physical and financial status, postoperative adjuvant therapy was not performed in all patients. Our results showed that acceptance of adjuvant therapy was greater in patients who were younger than 60 years old, had 12 or more resected LNs, and had stage pT3, or TNM stage IIIa/b (Table 1). Because this was a retrospective study, some patient details were not available for analysis. Additionally, there were differences in the individual dosages and targets of PORT, the schemes and number of cycles of POCT, and the percentages of patients who received PORT and/or POCT. We did not analyze these detailed differences. However, our multivariate Cox analysis indicated that adjuvant therapy was significantly and independently associated with improved OS, DFS, and LR. A third limitation is that we did not consider the impact of different surgical approaches; previous research indicated that the surgical approach may affect the survival rate of patients with pN+ ESCC [30, 31]. Finally, factors such as gender, TNM stage, and the number and presence of dissected LNs can also influence OS and DFS, but we did not analyze these variables. However, our subgroup analysis showed that regardless of the number of dissected LNs, adjuvant therapy improved OS, DFS, and LR (data not shown). Conducting randomized controlled trials that examine the impact of POCT and/or PORT on survival of patients with pN+ ESCC is difficult in our institution and in China generally. We therefore believe that studies of large samples from one or more high-volume institutions, such as ours, provide valuable new information.

## 5. Conclusions

In conclusion, we found that the long-term survival of patients with pN+ ESCC after R0 resection remains poor. However, our single-center retrospective study of these patients showed that after surgery (mainly through the left thoracic approach), postoperative adjuvant therapy—either POCT, PORT, or POCRT—significantly improved OS and DFS, and POCRT improved OS and DFS compared with POCT. Furthermore, PORT and POCRT reduced LR, but POCT had no impact on LR. These results indicate that adjuvant therapy—either POCT, PORT, or POCRT—should be strongly considered for patients with pN+ ESCC after R0 resection and that POCRT is preferred if the patient can tolerate it.

## Figures and Tables

**Table 1:** Clinical characteristics of patients who did and did not receive adjuvant therapy.

Factor	Group	Cases, *n*	Adjuvant therapy, *n* (%)				*p*
			None	POCT	PORT	POCRT	
Gender	Male	338	95 (28.1%)	168 (49.7%)	42 (12.4%)	33 (9.8%)	0.904
	Female	115	36 (31.3%)	54 (47.0%)	15 (13.0%)	10 (8.7%)	
Age (years)	≤60	247	55 (22.3%)	130 (52.6%)	30 (12.1%)	32 (13.0%)	0.001
	>60	206	76 (36.9%)	92 (44.7%)	27 (13.1%)	11 (5.3%)	
Site of lesion	Upper	40	14 (35.0%)	17 (42.5%)	8 (20.0%)	1 (2.5%)	0.458
	Middle	312	86 (27.6%)	155 (49.7%)	38 (12.2%)	33 (10.6%)	
	Lower	101	31 (30.7%)	50 (49.5%)	11 (10.9%)	9 (8.9%)	
Length of lesion (cm)	<6	219	73 (33.3%)	97 (44.3%)	26 (11.9%)	23 (10.5%)	0.144
	≥6	234	58 (24.8%)	125 (53.4%)	31 (13.2%)	20 (8.5%)	
Resected LNs, *n*	<12	264	88 (33.3%)	111 (42.0%)	42 (15.9%)	23 (8.7%)	0.00
	≥12	189	43 (22.8%)	111 (58.7%)	15 (7.9%)	20 (10.6%)	
pT stage	pT2	57	23 (40.4%)	19 (33.3%)	4 (7.0%)	11 (19.3%)	0.007
	pT3	367	96 (26.2%)	189 (51.5%)	51 (13.9%)	31 (8.4%)	
	pT4	29	12 (41.4%)	14 (48.3%)	2 (6.9%)	1 (3.4%)	
pN stage	pN1	294	90 (30.6%)	142 (48.3%)	38 (12.9%)	24 (8.2%)	0.143
	pN2	131	29 (22.1%)	72 (55.0%)	15 (11.5%)	15 (11.5%)	
	pN3	28	12 (42.9%)	8 (28.6%)	4 (14.3%)	4 (14.3%)	
pTNM stage	IIb	42	17 (40.5%)	16 (38.1%)	3 (7.1%)	6 (14.3%)	0.147
	IIIa	250	73 (29.2%)	120 (48.0%)	35 (14.0%)	22 (8.8%)	
	IIIb	108	21 (19.4%)	64 (59.3%)	13 (12.0%)	10 (9.3%)	
	IIIc	53	20 (37.7%)	22 (41.5%)	6 (11.3%)	5 (9.4%)	
Differentiation	Well/middle	331	93 (28.1%)	59 (48.4%)	8 (6.6%)	17 (13.9%)	0.035
	Poor	122	38 (31.1%)	163 (49.2%)	49 (14.8%)	26 (7.9%)	

CT: chemotherapy; CRT: chemoradiotherapy; LN: lymph node; RT: radiotherapy.

**Table 2:** Multivariate analysis of factors associated with OS.

Factor	HR (95% CI)	*p*
Gender		
Male	1.332 (1.041, 1.705)	0.023
Female	1 (ref)	
Site of lesion		
Upper	1.094 (0.713, 1.679)	0.681
Middle	1.368 (1.049, 1.782)	0.020
Lower	1 (ref)	
Resected LN, *n*		
<12	1.270 (1.021, 1.581)	0.032
≥12	1 (ref)	
pTNM stage		
IIb	1 (ref)	
IIIa	1.350 (0.898, 2.030)	0.149
IIIb	2.105 (1.359, 3.259)	0.001
IIIc	2.888 (1.797, 4.641)	<0.001
Adjuvant therapy		
None	1 (ref)	
POCT	0.604 (0.471, 0.774)	<0.001
PORT	0.543 (0.382, 0.771)	0.001
POCRT	0.389 (0.257, 0.590)	<0.001

**Table 3:** Multivariate analysis of factors associated with DFS and LR.

Factor	Disease-free survival		Locoregional-free survival	
	HR (95% CI)	*p*	HR (95% CI)	*p*
Site of lesion				
Upper	1.062 (0.696, 1.621)	0.781	1.074 (0.628, 1.836)	0.795
Middle	1.388 (1.074, 1.794)	0.012	1.406 (1.020, 1.938)	0.038
Lower	1 (ref)		1 (ref)	
pTNM stage				
IIb	1 (ref)		1 (ref)	
IIIa	1.342 (0.906, 1.988)	0.142	0.876 (0.571, 1.343)	0.543
IIIb	1.942 (1.271, 2.968)	0.002	1.300 (0.814, 2.078)	0.272
IIIc	2.682 (1.691, 4.253)	<0.001	1.870 (1.115, 3.135)	0.018
Adjuvant therapy				
None	1 (ref)		1 (ref)	
CT	0.664 (0.523, 0.843)	0.001	0.840 (0.627, 1.126)	0.244
RT	0.612 (0.431, 0.867)	0.006	0.490 (0.301, 0.797)	0.004
CRT	0.395 (0.263, 0.593)	<0.001	0.333 (0.190, 0.584)	<0.001

## Data Availability

The datasets used and/or analyzed during the current study are available from the corresponding author on reasonable request.
